# Promoting Elementary Pupils’ Learning Motivation in Environmental Education with Mobile Inquiry-Oriented Ambience-Aware Fieldwork

**DOI:** 10.3390/ijerph17072504

**Published:** 2020-04-06

**Authors:** Morris Siu-yung Jong

**Affiliations:** Department of Curriculum and Instruction & Centre for Learning Sciences and Technologies, The Chinese University of Hong Kong, Hong Kong, China; mjong@cuhk.edu.hk

**Keywords:** M-learning, environmental education, learning motivation, ambient learning, outdoor fieldwork, inquiry-oriented learning

## Abstract

Mobile learning (M-learning) has been in high regard for motivating today’s children to learn in schools. The present initiative, which harnesses M-learning in environmental education, aims to promote elementary pupils’ learning motivation through engaging them in conducting mobile inquiry-oriented ambience-aware fieldwork (MIAF) in outdoor landscapes. Besides presenting the rationale and pedagogic design of the initiative, this paper reports and discusses the findings of a quasi-experiment which examined the motivational effectiveness of MIAF in comparison with the conventional inquiry-oriented fieldtrip-based learning approach’s. The experiment was grounded upon the instructional motivation theory of ARCS (Attention, Relevance, Confidence and Satisfaction), involving a total of 145 elementary pupils. Results showed that, compared to the conventional approach, MIAF had significantly positive effects on the experimental group (versus the control group) upon the constructs of “A”, “C”, and “S”, but not “R”. This study offers researchers and practitioners in the domains of environmental education and M-learning new insights into adopting mobile devices in outdoor contexts, in particular, shedding light on designing and implementing inquiry-oriented fieldtrip-based learning in the natural environment.

## 1. Introduction

Pupils’ learning performance has a strong correlation with their learning motivation [1,2]. In fact, one of the major challenges that many teachers are facing in schools for years has still been “how to better motivate my pupils to learn” [3,4,5,6]. With the rapid development of and prevalent access to mobile technology in the last decade, harnessing mobile devices in the pedagogic process, particularly in the contexts of K–12 education, has been deemed to be a desirable strategy and a promising trend in motivating today’s “digital native” children to engagingly participate in education-related activities, regardless in formal or informal learning/teaching settings [7,8]. In general, “learning with mobile technology” is termed *mobile Learning* or *M-learning* [9,10]. As a subset of M-learning, *ambient learning* leverages, in particular, context-aware computing technology in the pedagogic process so that the learning materials can be dynamically adaptive to learners’ current ambient situations [11,12]. 

Environmental education is regarded as one of the most important key learning areas in basic education [13,14]. In general, the major objectives of environmental education are threefold: (i) to equip pupils with awareness and knowledge about the environment, (ii) to cultivate them with the attributes that form the basis of environmental citizenship, and (iii) to develop their skills for supporting environmental protection [15,16]. In Hong Kong elementary schools, the formal environmental education is included in a mandatory subject, namely *General Studies* [17]. In line with the government’s recent educational reforms [18], teachers of this subject are required to adopt diversified learning and teaching strategies in the pedagogic process for providing pupils with substantial self-directed and preferably inquiry-oriented, real-life learning opportunities beyond the classroom [19,20]. Under these circumstances, outdoor fieldtrip-based learning has become a popular approach for offering pupils so-called “individualized, authentic inquiry-based learning experiences” in General Studies [21,22]. Typically, paper-based worksheets are the only learning support for guiding pupils to accomplish inquiry tasks in the fieldtrips [23]. However, studies have unfolded, from the perspective of young learners, this kind of conventional worksheet-supported fieldwork is regarded as “boring learning”, e.g., [24,25]. 

The present initiative, namely “mobile inquiry-oriented ambience-aware fieldwork” (MIAF), aims to incorporate ambient learning in environmental education, in particular, employing the location-based context-aware computing technology, to support pupils in motivatedly carrying out inquiry-oriented fieldtrip-based learning in natural landscapes. Apart from presenting the rationale and pedagogic design of MIAF, this paper will report and discuss the results of a learning experiment underpinning Keller’s [26,27,28,29] instructional motivation theory for evaluating MIAF’s motivational effectiveness in comparison with the conventional inquiry-oriented fieldtrip-based approach’s. 

After this introduction, the rest of this paper is organised as follows. The next section will be a review of the related studies framing the present work. After that, the design and findings of the learning experiment will be delineated. The research limitations, future work, and implications will be discussed at the end of the paper.

## 2. Literature Review 

### 2.1. M-Learning and Ambient Learning

M-learning is commonly defined as the employment of mobile technology and devices, as well as other technologies (e.g., web and multimedia), to support the course of learning and teaching, regardless in formal or informal educational settings, inside or outside classrooms, and within indoor or outdoor contexts [9,10,30,31]. In the recent Horizon Report (K–12 Edition), M-learning is not only regarded as a desirable strategy, but also a prominent global trend for educators and teachers to frame and shape educational activities for today’s K–12 learners [32]. In Hong Kong, incorporating M-learning in schooling is also one of the government’s major technological strategies advocated in the current pedagogic and curricular reform in K–12 education [33,34]. 

As a genre of M-learning, ambient learning emphasises the importance of “instructional contextualisation”, i.e., in the course of learning, the learning content is dynamically adaptive to the ambience where a learner is currently situated [11,12]. From an educational perspective, ambient learning harnesses the pedagogic idea of “just-in-time learning”, which advocates the instant provision of need-based learning resources for co-workers in vocational settings when ones are encountering uncertainties or new problems [35]. Those “readily available” resources are aimed at efficiently filling in ones’ knowledge gaps in a just-in-time fashion. Usually, the resources are personalised based on ones’ instant learning needs, in the form of digital, self-contained pieces for easy access [36].

From a technological perspective, ambient learning leverages context-aware computing technology (CACT) which can proactively gather learners’ current contextual information (e.g., location, time, privilege level, etc.) and the dynamic changes of the information [11,12]. By analysing the collected information, personalised learning support can then be formulated and provided in accordance with ones’ ambient situations [35]. Usually, CACT is deployed via a technical system (or sub-system) with a combination of both software (e.g., a mobile App) and hardware (e.g., a mobile phone or tablet) [36]. For example, the Global Positioning System (GPS), which is a sort of CACT employing satellite-oriented radio-navigation for autonomously collecting and dissecting users’ location-based contextual information, can be deployed with, for example, a handheld mobile device [23]. 

### 2.2. EduVenture

*EduVenture* [37] (formerly named EagleEye in its prototyping stage [23]) is an open-access ambient learning system for educational researchers and practitioners to create digital GPS-supported ambience-aware materials for facilitating learners to conduct outdoor, self-directed fieldtrip-based learning. There are two core components in this system: the *Composer* and *Explorer*. The Composer is a computer-based software platform for authoring ambience-aware learning materials. The Explorer is a mobile application (i.e., an App) run on mobile devices (e.g., mobile phones or tablets) for accessing the materials created with the Composer. While the technical details of EduVenture are well described in the related literature [38], the following will highlight the pedagogic affordance of this system. 

**Figure 1:**
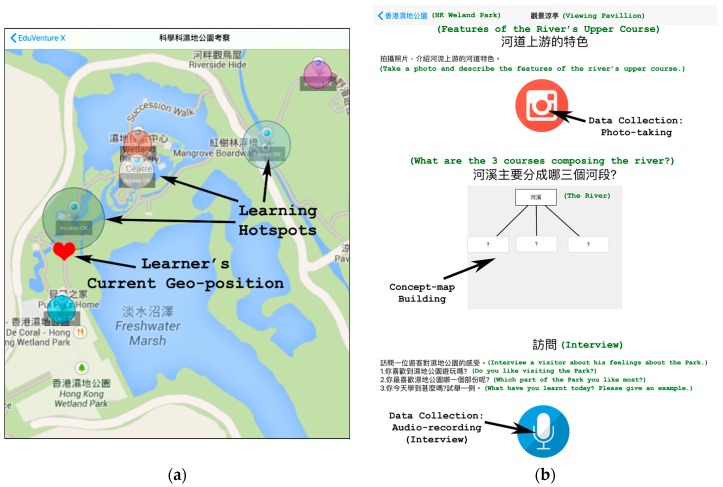
EduVenture: (**a**) First-tier Interface; (**b**) Second-tier Interface

The user interface of an ambience-aware learning material created with EduVenture is in a two-tier design. The first-tier interface is a digital map of the fieldtrip site (see Figure 1a). On the map, the heart-shaped icon denotes a learner’s current geo-position in the real world, in accordance with the GPS signal received by the learner’s mobile device. While the learner moves from one point to another, the icon on the map will move synchronously. When the learner physically reaches a designated “learning hotspot” (i.e., a circle-shaped spot on the map), the second-tier interface will be triggered, and the “learning anchors” embedded in that hotspot will pop up to guide the learner to accomplish the pre-set learning tasks therein (see Figure 1b). The anchors can be in the forms of audio-/video-based introductory briefing, concept-map building, various data collection exercises (via photo-taking or audio-/video-recording), etc. When the learner leaves that hotspot, the second-tier interface will be automatically swapped to the first-tier interface again. 

There have been several research instances of adopting EduVenture in secondary education, as well as tertiary education, but it has not yet been adopted in elementary education. For example, it has been used in humanities education to support middle- and high-school students in studying societal issues, such as “to what extent are traditional customs compatible with modern society” and “how people react differently to the opportunities and challenges brought by globalization”, e.g., [23,24]. This system has also been used in cultural education to support university students in investigating various practices of heritage preservation and conservation, e.g., [39]. In the present study, EduVenture was adopted to develop the ambience-aware learning material used in the experimental manipulation in the learning experiment (see Section 3.2). It is the first study harnessing this system in elementary education and, in particular, focusing on environmental education. 

### 2.3. Environmental Education in Hong Kong Elementary Schools

General Studies (hereafter referred to as GS) is one of the mandatory subjects in Hong Kong elementary education, constituting about 12% to 15% of the overall schooling time [17]. It is an integrated subject combining the disciplines of Social Science, Science, and Technology. In line with the recent education reform of “Learning to Learn 2.0” [18], the curriculum of GS has been revamped with the aim of better equipping pupils with knowledge, skills and attitudes for understanding and empathising the micro, macro and dynamic changes in various local, regional as well as global contexts [19]. The formal elementary environmental education in Hong Kong is embedded in GS. 

One of the major educational objectives of GS is to develop pupils’ curiosity and interest in the natural world, and cultivate their care and concern for the environment. “People and Environment” is one of the six core study areas in the new GS curriculum [17]. Upon the curricular framework, in each area, there are several thematic learning modules in accordance with each grade level. For example, “Love of Nature” is one of the modules covered in Grade 5 in the area of “People and Environment”. The module aims to arouse pupils’ environmental awareness, equipping them with basic knowledge about the characteristics of natural landscapes (e.g., wetlands, geo-parks) and fostering their care for the natural environment. The design of the learning experiment in the present study hinges on this module. 

The government’s statutory policy spells out that approximately 20% of learning activities in GS should be in a self-directed manner, preferably inquiry-based [20]. In addition, schools are strongly encouraged to make good use of (i) real-life, outdoor contexts to widen pupils’ scope of learning beyond the classroom, and (ii) technology to support them in carrying out self-directed learning. In fact, the recent meta-reviews, e.g., [21,22], of the new GS curriculum have also seconded the importance of (i) providing pupils with authentic inquiry opportunities for promoting their motivation in studying the environmental components, and (ii) harnessing technology in learning and teaching in this subject. 

In a recent study of the implementation of the new GS curriculum [25], it has been revealed that teachers do adopt some M-learning strategies (e.g., Nearpod^®^, Socrative^®^, Google Classroom^®^, Apple Classroom^®^) to support the learning and teaching activities inside the classroom; however, they lack clues as to how to facilitate their pupils to conduct M-learning outside the classroom. On the other hand, teachers do organise outdoor fieldtrips for pupils to conduct inquiry tasks in accordance with the environmental components in the curriculum. However, the self-directed learning scaffolds provided to pupils are usually in the form of traditional paper-based worksheets, without taking the pedagogic advantages that technology can introduce. Pupils’ hand-writing and quick-sketching are the usual means to respond to the scaffolds and/or show their accomplishment of the inquiry tasks. The study also indicated that pupils were unmotivated to participate in this kind of conventional worksheet-supported fieldtrip, which is in agreement with several earlier studies reviewing or evaluating the pedagogic effectiveness of conventional fieldwork in the settings of school education, e.g., [23,24,38,40].

### 2.4. Inquiry-Oriented Learning

Learner-centred learning involves a constructivist process in which learners actively construct knowledge of their own, instead of passively receiving it from others [41,42,43,44]. *Inquiry-oriented learning*, which is also known as inquiry- or enquiry-based learning, is one of the popular learner-centred instructional approaches being advocated in K–12 education [31,40]. It emphasises learners’ active participation and responsibility for exploring phenomena and developing knowledge [45,46]. However, in the course of inquiry-oriented learning, adequate scaffolds should be provided for learners, especially for young school learners; otherwise, they may not be able to accomplish the required inquiry tasks and achieve the designated learning goals, and eventually will be largely frustrated by the learning process [3,5,24]. 

Through examining the related literature published in the past two decades, Pedaste et al. [47] generalised a scaffolding framework for inquiry-oriented learning. The self-directed part of this framework involves four major inquiry anchors: *orientation*, *investigation*, *conclusion*, and *reflection*. The orientation anchor is to provide learners with background information about the topic to be inquired and connect their related prior knowledge to the topic. The investigation anchor is to offer learners clues for collecting and scrutinising new information in order to accomplish the inquiry tasks involved in the learning process. The conclusion anchor is to support learners in expressing and presenting the findings corresponding to the inquiry tasks. The reflection anchor is to empower learners to review their achievement of the learning goals, as well as their strengths and weaknesses in the inquiry process. In the present study, Pedaste et al.’s self-directed scaffolding framework for inquiry-oriented learning was adopted to develop the learning materials used in both experimental and control manipulations in the learning experiment (see Section 3.2).

### 2.5. Instructional Motivation Theory of ARCS 

“How to motivate learners to learn” is always a vital pedagogic consideration in any kinds of instructional strategies and educational settings [3,5,41,42]. “Motivation”, which is one of the most important conceptions in the realm of cognitive science for interpreting human behaviours [4], is “the attribute of moving [people] to do or not to do something” [1], i.e., “what they desire, what they choose to do, and what they commit to doing” [29].

Since the early 1970s, “learning motivation” has been conceptualised and theorised by several renowned researchers, e.g., [48,49,50], from different perspectives in different learning and teaching settings. With respect to instructional design, one of the most well-developed theoretical models is Keller’s instructional motivation theory of ARCS—an acronym that stands for “Attention”, “Relevance”, “Confidence”, and “Satisfaction” [26,27,28,29]. 

Keller [26,27] argued that the key to motivating pupils to learn was to arouse and sustain their curiosity in a learning environment where (i) the learning content should be relevant to their personal interest, and (ii) the learning process should be instrumental in scaffolding them to achieve their desired goals. Through holistically and rigorously reviewing on literature related to the conceptions and theories of learning motivation, Keller synthesised a theoretical model, ARCS, that conceptualises instructional motivation with four categorical constructs. A—Attention refers to how well an instructional approach can capture learners’ interest, stimulate their thinking, and sustain their attention throughout the pedagogic process. R—Relevance refers to how well an instructional approach can address learners’ needs, set meaningful learning goals for them, and offer them learning experience related to the real world. C—Confidence refers to how well an instructional approach can help learners develop a positive expectation of success, support them in believing their ability to achieve the learning goals, and let them know the final success stemming from their effort made. S—Satisfaction refers to how well an instructional approach can facilitate learners to recognise the rewards of the newly acquired knowledge, articulate positive feelings to their interim accomplishments, and strengthen their sense of success at the end of the pedagogic process.

Grounded on the theoretical foundation of ARCS, Keller [28,29] further constructed and validated a 36-item questionnaire-based quantitative instrument, namely “Instructional Materials Motivation Survey” (IMMS), for measuring learners’ motivational reactions to self-directed instructional materials, with respect to the four constructs. The overall reliability estimate (Cronbach’s alpha) of the instrument is 0.96, while the reliability estimates of the four constructs are between 0.81 and 0.92. In fact, the ARCS model and IMMS instrument have been widely employed in various learning and teaching settings (including conventional classrooms, e.g., [51] and E-learning environments, e.g., [52]), as well as at different education levels (including school education, e.g., [53] and tertiary education, e.g., [54]).

In a recent classroom-based M-learning study, Yang [55] adopted the ARCS model to evaluate the motivational effectiveness of the use of head-mounted display VR (virtual reality) to support high-school learners in conducting self-directed learning in Geography lessons. In the study, based on Keller’s original work on IMMS [28,29], Yang developed and validated a customised version of the instrument for M-learning. The revised IMMS consisted of a total of 20 items, with every five items contributing to one of the four constructs. The revision was based on the research context of her study, involving (i) customising the original wording of each IMMS item, and (ii) discarding some items irrelevant to M-learning to strengthen the model fit. The overall reliability estimate (Cronbach’s alpha) of the revised IMMS is 0.97, while the reliability estimates of the four constructs are between 0.82 and 0.90. In the present study, a further customised version of Yang’s 20-item IMMS instrument was composed (see Section 3.5)

## 3. Method

The present study aimed to evaluate the effectiveness of the initiative of harnessing ambient learning to promote elementary pupils’ learning motivation in conducting inquiry-oriented outdoor environmental fieldwork. This initiative is termed “mobile inquiry-oriented ambience-aware fieldwork” (MIAF) in this paper. 

### 3.1. Participants

The learning experiment was conducted in a public elementary school located in an urban district in Hong Kong, involving all Grade-5 pupils of the school (n = 148). All of them had prior experience in using mobile devices (mobile phones/tablets). Before the experiment, the pupils were ordered in accordance with their GS examination scores in the previous semester and then assigned alternately to either the experimental group (n = 74) or the control group (n = 74).

### 3.2. Experiential and Control Manipulations

This learning experiment involved conducting self-directed fieldwork for studying a thematic module of GS, “Love of Nature”, in which the participants were required to look into the characteristics of the Hong Kong Wetland Park and reflect on the importance of caring for the natural environment [19]. The experimental manipulation was MIAF, while the control manipulation was “conventional inquiry-oriented worksheet-supported fieldwork” (CIWF). The design of the self-directed learning materials for both MIAF and CIWF was grounded on Pedaste et al.’s [47] scaffolding framework of inquiry-oriented learning. The MIAF material was created with EduVenture [37], while the CIWF material was in the form of paper-based worksheets (i.e., the conventional approach, as described in Section 2.3). 

Both manipulations covered the same number of designated “learning hotspots” at the fieldtrip site. Each hotspot contained the same number of inquiry anchors that embodied the inquiry sub-tasks of orientation, investigation, conclusion, and reflection [47]. As for the MIAF material, it was accessible through a mobile phone or tablet, which possessed the GPS-enabled ambience-aware feature. The experimental-group participants were required to respond to the inquiry anchors (i.e., to complete the inquiry sub-tasks at every hotspot “digitally” via the first- and second-tier interfaces as described in Section 2.2). For example, the orientation anchors were presented in the form of video-based introduction, the investigation anchors were presented in the form of data collection work via photo-taking or audio-/video-recording, the conclusion anchors were presented in the form of concept-map development, and the reflection anchors were presented in the form of audio-recorded self-reporting. Regarding the CIWF material, it was conventionally text- and paper-based, and the control-group participants were required to respond to the inquiry anchors through hand-writing and/or quick-sketching.

### 3.3. Research Review Panel

A research review panel was set up with a combination of five persons, including a GS educator in the Faculty Education from another local university, two senior GS teachers (with 10 and 14 years, respectively, of teaching experience) from the participating schools, and two senior GS teachers (with 12 and 13 years, respectively, of teaching experience) from two different non-participating elementary schools. The panel was responsible for reviewing the design of both experimental and control manipulations, in particular, examining whether the materials (i) aligned with the GS’s curricular aim, and (ii) were with comparable quality. In addition, the panel reviewed the quantitative data collection instrument (i.e., the IMMS questionnaire) used in the present study (see Section 3.5) and gave suggestions for revising the wording of some questionnaire items to secure that the participants (Grade-5 pupils) were able to literally understand the items.

### 3.4. Research Procedures

The experimental and control manipulations were conducted separately, but were administered with the same research procedures. Each manipulation, together with the data collection work, was completed within three consecutive days, involving the following steps:

- Introduction (Day 1). A 30-minute introductory session was conducted in the classroom for briefing the experimental group/control group on how to use the MIAF/CIWF material in the fieldtrip, as well as the logistical arrangement and some safety matters.
- Fieldwork (Day 2). The duration of the fieldtrip was 3 hours (excluding the transportation time). The experimental-group participants conducted MIAF, while the control-group participants conducted CIWF.
- Data Collection (Day 3). A questionnaire-based IMMS (see Section 3.5) was conducted in the classroom. Each group was given 25 minutes to complete the survey. After the survey, three participants from each group were selected on a random basis to participate in a one-hour semi-structured group interview (see Section 3.5).

### 3.5. IMMS Questionnaire and Interview Questions

The IMMS questionnaire was written in Chinese (i.e., the mother language of the participants). It consisted of 20 items which were customised from Yang’s [55] work. Each group of five items built one of the ARCS constructs (Attention, Relevance, Confidence, and Satisfaction). The customisation was based on the pedagogic and curricular settings of the present study. Nine items of the first customised version of the questionnaire were further paraphrased after receiving the comments from the research review panel. In addition, six Grade-5 pupils from two non-participating schools were invited to cross-check each item’s wording for securing that the final version of the questionnaire was understandable to Grade-5 pupils. In the administered version of the questionnaire, the 20 items were placed in random order, with each item along with a 7-point symmetric Likert scale from “1: Strongly Disagree” to “7: Strongly Agree”. In order to ensure a higher return rate, the survey was conducted an anonymous basis [56], i.e., no participants’ personal information was collected. The translated and categorised version of the questionnaire is shown in the Appendix of this paper. 

The collection of qualitative data via the interviews (in Chinese) was aimed at supplementing the IMMS findings. The same open-ended interview questions were used in the two semi-structured group interviews (one for the experimental group and one for the control group). The questions centred on the four constructs of the ARCS model and the context of the present study, including:

- Could yesterday’s fieldtrip stimulate your learning interest in the natural environment?
- Was the environmental knowledge gained in the fieldtrip valuable to your daily life?
- How effective was the self-directed learning material for facilitating you to conduct the fieldwork?
- Did you feel rewarded after accomplishing the fieldwork?

## 4. Findings

There were three pupils in total who were excluded in the data collection exercise. One of them was absent in the introduction session (on Day 1), and two of them were absent from the fieldtrip (on Day 2). Hence, the total number of survey subjects was 145; 72 from the experimental group and 73 from the control group. A total of 145 fully completed IMMS questionnaires were received. The response rate was 100%. 

The overall reliability estimate (Cronbach’s α) of the IMMS instrument is 0.91. The reliability estimates of the four ARCS constructs are between 0.79 and 0.87 (see Table 1). The values of 20 item-total correlations (ITC) corresponding to each of the 4 ARCS constructs are between 0.78 and 0.90, indicating that the data’s reliability is satisfactory. In addition, the confirmatory factor analysis (CFI = 0.91, GFI = 0.92, RMR = 0.08 and SRMR = 0.07) shows that each of the 20 items retains in the originally assigned ARCS construct (see Table 2). The comparative analyses of the experimental and control groups’ learning motivation corresponding to the four constructs are reported in the following sections.

### 4.1. Attention

Table 3 shows the descriptive statistics of the Attention construct corresponding to the experimental and control groups. An independent samples *t*-test on the two average scores reveals that the experimental group’s “Attention” (5.42) was significantly higher than the control group’s (4.11), *t*(143) = 5.51, *p* < 0.001, with Cohen’s *d* = 0.92, i.e., a large effect size. In the two semi-structured group interviews with the three experimental-group pupils (pseudonyms: *Eason*, *Ella*, and *Erwin*) and three control-group pupils (pseudonyms: *Candy*, *Charles*, and *Crystal*), they shared their motivational feelings related to the Attention construct. Table 4 displays the related interview excerpts translated into English. 

### 4.2. Relevance

Table 5 shows the descriptive statistics of the Relevance construct corresponding to the experimental and control groups. An independent samples *t*-test on the two average scores reveals that there was no significant difference between the experimental group’s “Relevance” (5.20) and the control group’s (4.98), *t*(143) = 0.79, *p* > 0.05, with Cohen’s *d* = 0.13, i.e., a tiny effect size. In the semi-structured group interviews with the experimental- and control-group interviewees, they shared their motivational feelings related to the Relevance construct. Table 6 displays the related interview excerpts translated into English. 

### 4.3. Confidence

Table 7 shows the descriptive statistics of the Confidence construct corresponding to the experimental and control groups. An independent samples *t*-test on the two average scores reveals that the experimental group’s “Confidence” (5.49) was significantly higher than the control group’s (4.51), *t*(143) = 4.03, *p* < 0.001, with Cohen’s *d* = 0.67, i.e., a medium effect size. In the semi-structured group interviews with the experimental- and control-group interviewees, they shared their motivational feelings related to the Confidence construct. Table 8 displays the related interview excerpts translated into English. 

### 4.4. Satisfaction 

Table 9 shows the descriptive statistics of the Satisfaction construct corresponding to the experimental and control groups. An independent samples *t*-test on the two average scores reveals that the experimental group’s “Satisfaction” (5.57) was significantly higher than the control group’s (4.32), *t*(143) = 5.41, *p* < 0.001, with Cohen’s *d* = 0.90, i.e., a large effect size. In the semi-structured group interviews with the experimental- and control-group interviewees, they shared their motivational feelings related to the Satisfaction construct. Table 10 displays the related interview excerpts translated into English. 

## 5. Further Discussion

According to the results of the learning experiment, MIAF had positive motivational effects on the experimental group upon all four motivational constructs of ARCS (with the average scores ranging from 5.20 to 5.57). Moreover, except for the Relevance construct, the motivational effectiveness of MIAF was significantly stronger than CIWF’s in the constructs of Attention (with a large effect size), Confidence (with a medium effect size), and Satisfaction (with a large effect size). 

“Attention” in Keller’s instructional motivation theory of ARCS refers to how well an instructional strategy can capture learners’ interest and maintain their attention throughout the learning process [26,27]. Therefore, developing pupils’ curiosity and sustaining their active participation are always crucial acts for keeping them in deep concentration on learning [1,3]. Featuring the GPS-enabled ambience-aware property, MIAF stimulated the experimental-group participants’ curiosity through the dynamic, pop-up learning scaffolds, see the excerpts of Eason and Erwin in Table 4 (cf. the static learning scaffolds in CIWF, see the excerpts of Candy and Charles). Additionally, at every learning hotspot, MIAF leveraged various multimedia means to make the experimental-group participants stay focused on accomplishing the required inquiry sub-tasks of orientation, investigation, conclusion and reflection [47], see the excerpts of Ella and Erwin in Table 4 (cf. the text-based learning scaffolds in CIWF, see the excerpts of Candy and Crystal). 

“Relevance” in Keller’s ARCS theory refers to how well an instructional strategy can provide learners with authentic learning experiences underpinning meaningful learning goals [26,27]. In fact, this idea aligns with the premise of many constructivist learning theories, e.g., [3,41,42] and approaches, e.g., [57,58,59,60] that learners are more willing to learn when they are situated in a real-world context to pursue the learning goals which are meaningful to their daily lives. In the experiment, both MIAF and CIWF authentically situated the experimental and control groups in the same authentic learning environment (the Hong Kong Wetland Park) to pursue the same meaningful learning goals (exploring the wetland’s characteristics and reflecting on the importance of caring for the natural environment), see the excerpts of Eason, Ella, Erwin, Candy, Charles and Crystal in Table 6. This can explain the finding of “no significant difference” between the motivational effectiveness of MIAF and CIWF in the “Relevance” construct. 

“Confidence” in Keller’s ARCS theory refers to how well an instructional strategy can help learners develop a positive expectation of success and support them in believing their ability to achieve the learning goals [26,27]. Usually, learning motivation will be promoted when pupils regard themselves are able to succeed in mastering the learning tasks [2,4,24,33]. Therefore, in the learning process, it is desirable to stimulate and reinforce their feelings of “personal control” and “expectancy for success” [3,5,28]. Leveraging the digital map featured with the GPS-enabled ambience-aware property, MIAF made the experimental-group participants have the feelings of “success” because of the consciousness of reaching the right locations for conducting the learning tasks, see the excerpts of Eason and Ella in Table 8 (cf. the control-group participants’ anxious feelings experienced in CIWF, see the excerpt of Candy). Also, MIAF empowered the experimental-group participants, at every learning hotspot, to efficiently document their collected data, conclusive expression and reflective report for accomplishing the investigation, conclusion and reflection sub-tasks, see the excerpts of Ella and Erwin in Table 8 (cf. the frustrations encountered by the control-group participants, see the excerpts of Charles and Crystal).

“Satisfaction” in Keller’s ARCS theory refers to how well an instructional strategy can facilitate learners to recognise the rewards of the newly acquired knowledge and strengthen their sense of formative and summative success throughout the learning process [26,27]. It is believed that pupils’ learning motivation will be reinforced if they experience satisfying outcomes to the learning task and perceive that the amount of work involved in the task is reasonable [24,48,49]. In the experiment, MIAF was able to foster the feelings of “rewarding”, “enjoyable”, and “a great success” among the experimental-group participants during the fieldtrip, see the excerpts of Eason, Ella and Erwin in Table 10 (cf. the unpleasurable feelings of “time-consuming”, “very tired”, and “not successful” experienced by the control-group participants, see the excerpts of Candy, Charles and Crystal).

## 6. Limitations and Future Work

Regarding the findings of the present study, it should be underlined that all participants were from an urban area of Hong Kong (see Section 3.1). Like other “digital native” kids around the world [7,8], they are in touch with mobile technology and devices in their daily lives. Thus, the experimental-group pupils were able to quickly get themselves familiarised with the operation of the App (i.e., EduVenture’s Explorer) without any difficulties during the introductory briefing session (see Section 3.4). If the experimental group (e.g., pupils from rural areas) had not possessed any previous mobile-device experience, some pre-training work would need to have been conducted for equipping them with the necessary operational knowledge prior to the learning experiment. 

Another research challenge is related to whether the desirable motivational learning effects of MIAF on the experimental group can be sustainable. In fact, there is a need for further investigation into the sustainability of the effects when the experimental-group pupils learn with MIAF again in studying other thematic modules related to environmental education in the GS curriculum. Moreover, it is worth further investigating the possibility of harnessing MIAF in other elementary educational activities in those subjects (e.g., historical and cultural education, social and humanities education, arts education, etc.) that involve curricular topics favourable to being studied in outdoor learning contexts.

## 7. Conclusions

Environmental education is one of the most important learning domains in fundamental education [13,14]. For ages, pupils’ weak motivation towards learning has been one of the persistent problems in school education [1,2,5,41,42]. In the last decade, M-learning has been regarded as a promising tactic or motivating today’s children to actively participate in school activities [9,10,31,32]. The present initiative, MIAF, integrates M-learning (in particular, ambient learning) into outdoor environmental fieldwork within the paradigm of self-directed, inquiry-oriented learning. In the learning experiment on evaluating its motivational effectiveness, MIAF had positive effects on the experimental group with respect to the four ARCS motivational constructs. Moreover, according to the results of the comparative analyses, except for the Relevance construct, MIAF significantly outperformed CIWF in the constructs of Attention, Confidence, and Satisfaction.

This paper offers educational researchers and instructional designers new insights into employing M-learning in outdoor contexts, in particular, shedding light on designing and implementing fieldtrip-based learning in natural landscapes. The involved pedagogic work is the first research instance that adopts the open ambient learning system, EduVenture, in elementary education, and in particular, environmental education. It is also the first research instance that incorporates the self-directed proposition of Pedaste et al.’s [47] inquiry-oriented learning model into ambient learning for supporting outdoor environmental fieldwork. With necessary wording customisation, M-learning researchers can make use of the revised IMMS questionnaire (with adequate reliability and construct validity), which was developed for the learning experiment discussed in this paper (see the Appendix), to evaluate their new M-learning or ambient learning initiatives in the aspect of learners’ motivation. 

The new GS curriculum requires elementary schools in Hong Kong to introduce diversified educational and technological strategies to provide pupils with more real-life, inquiry-based learning opportunities and support them in becoming self-directed learners [17,18,19,20]. GS teachers have been exploring effective ways to implement the new curriculum; nevertheless, they lack inspirations on how to leverage mobile technology to facilitate pupils’ learning outside the classroom [21,22,25]. The present work provides the related practitioners with a theoretically grounded, empirically proved reference for scaffolding pupils to motivatedly pursue self-directed, inquiry-oriented fieldwork in the natural environment. 

## Figures and Tables

**Table 1:** Reliability evaluation of the IMMS data corresponding to the ARCS model.

Attention (α = 0.87)		Relevance (α = 0.81)		Confidence (α = 0.79)		Satisfaction (α = 0.80)	
**Item**	**ITC**	**Item**	**ITC**	**Item**	**ITC**	**Item**	**ITC**
A1	0.90	R1	0.81	C1	0.78	S1	0.82
A2	0.81	R2	0.80	C2	0.81	S2	0.79
A3	0.87	R3	0.79	C3	0.79	S3	0.80
A4	0.85	R4	0.84	C4	0.82	S4	0.79
A5	0.89	R5	0.79	C5	0.79	S5	0.82

**Table 2:** Factor loadings corresponding to the ARCS model.

Item	Factor Loading			
	Attention	Relevance	Confidence	Satisfaction
A1	0.78			
A2	0.80			
A3	0.79			
A4	0.81			
A5	0.83			
R1		0.77		
R2		0.81		
R3		0.78		
R4		0.75		
R5		0.79		
C1			0.79	
C2			0.75	
C3			0.76	
C4			0.80	
C5			0.85	
S1				0.77
S2				0.78
S3				0.80
S4				0.79
S5				0.72

**Table 3:** Descriptive statistics: Attention.

	Experimental Group (MIAF) (n = 72)	Control Group (CIWF) (n = 73)
Average	5.42	4.11
Standard Deviation	1.36	1.50

**Table 4:** Interview excerpts: Attention.

Group	Excerpts
Experimental Group (MIAF)	Eason: I was very engaged yesterday … the just-in-time pop-up learning tasks shown on the screen could effectively alert me to what I had to pay attention to during the fieldtrip. Ella: The multimedia features of the App made me more attentive to some subtle details of the fieldwork that could have been overlooked easily. Erwin: Seeing the heart-shaped icon moving on the screen in accordance with my movement, as well as looking forward to receiving the next sudden pop-up task, made the whole fieldtrip more exciting.
Control Group (CIWF)	Candy: The worksheets were too wordy … I do not like reading too many texts. Charles: I cannot regard yesterday’s fieldtrip as a very interesting learning activity … the worksheets looked boring. Crystal: The worksheets were not effective for helping me know whether I had reached the right location [hotspot] for conducting the required learning tasks.

**Table 5:** Descriptive statistics: Relevance.

	Experimental Group (MIAF) (n = 72)	Control Group (CIWF) (n = 73)
Average	5.20	4.98
Standard Deviation	1.56	1.61

**Table 6:** Interview excerpts: Relevance.

Group	Excerpts
Experimental Group (MIAF)	Eason: I learned many natural characteristics of the Hong Kong Wetland Park. I think most of the other wetlands in Hong Kong or in other places share similar characteristics. Ella: After the fieldtrip, I had a deeper feeling about the importance of environmental protection for human beings. Erwin: The knowledge gained in yesterday’s fieldtrip is profound in my head.
Control Group (CIWF)	Candy: I learned some useful tips for green living on the fieldtrip. Charles: Yesterday’s fieldwork was important for me to learn the related environmental knowledge. Crystal: The fieldwork did make me reflect on what and how my family members and I should do more in order to better protect our natural landscapes.

**Table 7:** Descriptive statistics: Confidence.

	Experimental Group (MIAF) (n = 72)	Control Group (CIWF) (n = 73)
Average	5.49	4.51
Standard Deviation	1.41	1.53

**Table 8:** Interview excerpts: Confidence.

Group	Excerpts
Experimental Group (MIAF)	Eason: With the heart-shaped “avatar” instantaneously indicating my geo-location at the fieldtrip site, I knew that I was on the right track to accomplish the required learning tasks. Ella: The step-by-step, pop-up inquiry hints could support me in conducting the learning tasks efficiently. Erwin: With the App … through photo-taking and audio-recording … all the data collection tasks could be carried out conveniently.
Control Group (CIWF)	Candy: During the fieldtrip, I was often uncertain about if I had arrived at the right location to do the right learning tasks. Charles: I found it was difficult for me to precisely write down my learning reflection in words on the worksheets … I am still an elementary pupil … often, I do not know how to write the words that I speak. Crystal: I am not good at drawing … I did not do well in the learning tasks that required quick-sketching.

**Table 9:** Descriptive statistics: Satisfaction.

	Experimental Group (MIAF) (n = 72)	Control Group (CIWF) (n = 73)
Average	5.57	4.32
Standard deviation	1.32	1.44

**Table 10:** Interview excerpts: Satisfaction.

Group	Excerpts
Experimental Group (MIAF)	Eason: The App gave me a rewarding experience … I wish I could use this App again for learning in the next environmental fieldtrip, haha … Ella: Yesterday’s fieldtrip was an enjoyable outdoor learning experience. Erwin: The fieldwork was a great success. I did learn a lot in the fieldwork, in particular, the ecological value of the wetlands in Hong Kong.
Control Group (CIWF)	Candy: I did not have enough time for completing all required learning tasks … during the fieldtrip, it was so time-consuming to write down all the findings and my reflection on the worksheets. Charles: I felt very tired after the fieldtrip … too many things to write down and draw on the worksheets. Crystal: I was not successful in finishing all required tasks in yesterday’s fieldwork … I was unable to draw my observations precisely on the worksheets, though I tried my best.

## Appendix: IMMS Questionnaire

**Construct**	**Item** *Please respond to the following questions based on your learning experience in yesterday’s fieldtrip:*	
Attention	A1	I stay in deep concentration on conducting the fieldwork.
	A2	The fieldwork stimulates my curiosity about the natural environment.
	A3	The self-directed fieldwork material is eye-catching.
	A4	I have surprising feelings when conducting the fieldwork.
	A5	The self-directed fieldwork material helps me stay focused on accomplishing the required learning tasks.
Relevance	R1	The learning experience gained in the fieldtrip is useful.
	R2	I can articulate my observations in the fieldtrip with the things I encounter in my daily life.
	R3	Participating in the fieldtrip is important for me to learn the related environmental knowledge.
	R4	The fieldwork helps me reflect on the importance of protecting the natural environment.
	R5	I can use the knowledge gained in the fieldtrip in the future.
Confidence	C1	During the fieldtrip, I realise that I can complete the required learning tasks without much difficulty.
	C2	The self-directed learning material makes me more confident in completing the fieldwork.
	C3	I feel confident that I can learn what I am supposed to learn regarding the natural environment through the fieldtrip.
	C4	I am able to carry out the required learning tasks competently in the fieldtrip.
	C5	The self-directed learning material supports me in completing the required learning tasks effectively.
Satisfaction	S1	Accomplishing the fieldwork gives me a satisfying feeling of achievement.
	S2	I am looking forward to participating in the next environmental fieldtrip.
	S3	I enjoy accomplishing the required learning tasks in the fieldtrip.
	S4	The fieldwork experience is pleasurable.
	S5	I feel rewarded for my effort at the end of the fieldtrip.
